# Supplementary material for: Altered microRNA Transcriptome in Cultured Human Liver Cells upon Infection with Ebola Virus
Source: Int J Mol Sci. 2021 Apr 6;22(7):3792. doi: 10.3390/ijms22073792 (PMC8038836; doi:10.3390/ijms22073792)
Supplement: Supplementary file 1 [file ijms-22-03792-s001.zip › Supplementary_File/D_ KEGG_Analysis_Results/16-30nt_pathway_Reston-96h-Huh7_vs_Control-96h-Huh7_up.mature_mirna_targets/hsa_GeneRatioDotPlot.pdf]

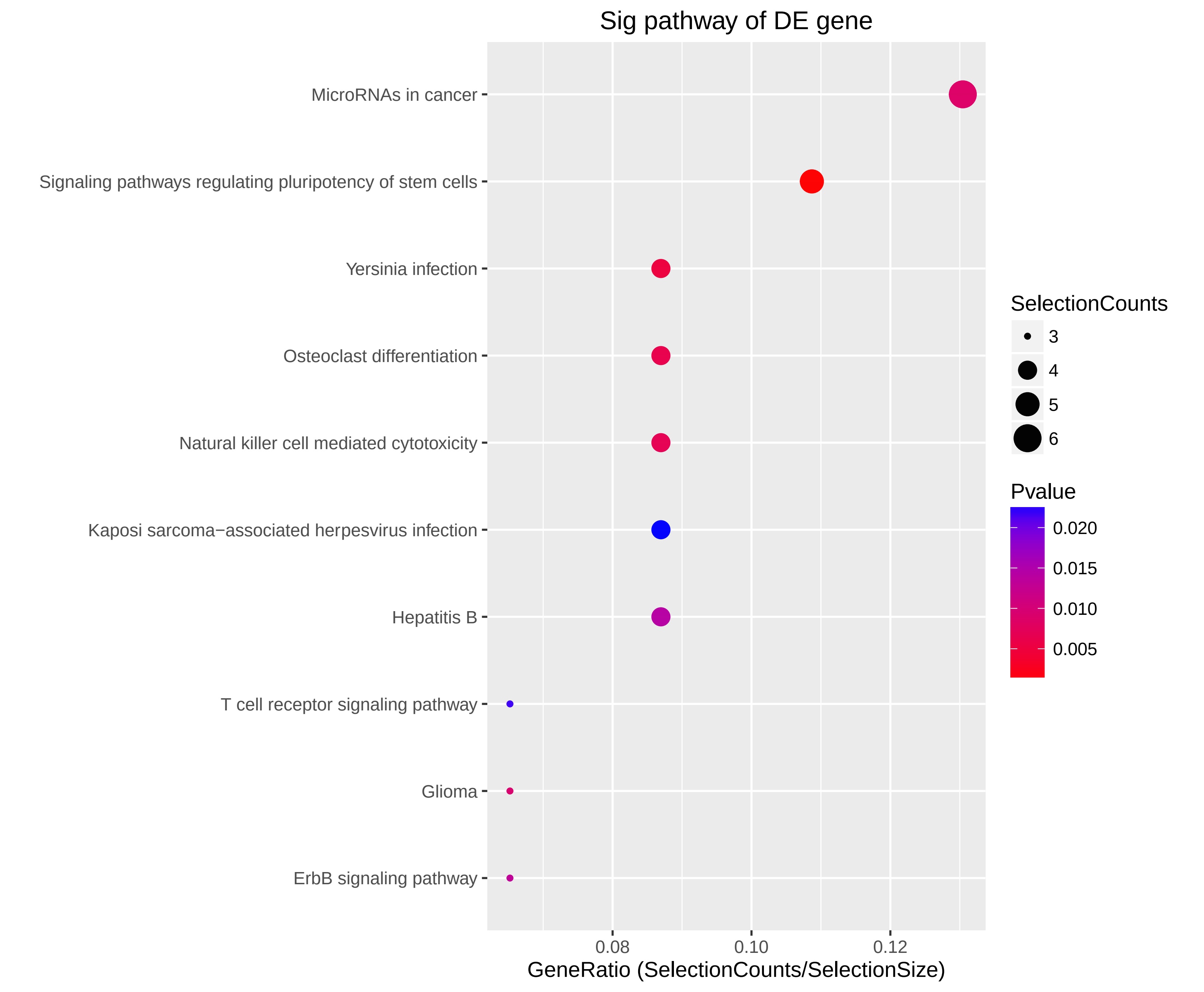

Sig pathway of DE gene
MicroRNAs in cancer
Signaling pathways regulating pluripotency of stem cells
Yersinia infection
Osteoclast differentiation
Natural killer cell mediated cytotoxicity
Kaposi sarcoma−associated herpesvirus infection
Hepatitis B
T cell receptor signaling pathway
Glioma
ErbB signaling pathway
0.08
0.10
0.12
GeneRatio (SelectionCounts/SelectionSize)
SelectionCounts
3
4
5
6
Pvalue
0.020
0.015
0.010
0.005
